# The scissors sign: a provocative test for detecting the leading-edge tear of subscapularis tendon: a diagnostic study

**DOI:** 10.1186/s12891-022-05621-1

**Published:** 2022-07-16

**Authors:** Sung-Min Rhee, Seung-Min Youn, Joon Hong Park, Geun Wu Chang, Yong Girl Rhee

**Affiliations:** 1 Shoulder & Elbow Clinic, Department of Orthopaedic Surgery, College of Medicine, Kyung Hee University Hospital, 23, Kyungheedae-ro, Dongdaemun-gu, Seoul, Republic of Korea; 2 The Department of Orthopaedic Surgery, Royal Brisbane and Women’s Hospital, Butterfield St, Herston, Queensland 4029 Australia; 3 Shoulder & Elbow Clinic, Department of Orthopaedic Surgery, Myongji Hospital, 697-24 Hwajung-dong, Deokyang-gu, Gyeonggi-do Goyang-si, South Korea

**Keywords:** Subscapularis tendon, Leading-edge tear, Physical examination, Provocation test, Scissors sign

## Abstract

**Background:**

Several physical examination tests and signs have been described to aid in the diagnosis of subscapularis (SSC) tear, but have limitations and variable sensitivity. This study aimed to introduce a novel test for detecting a leading-edge tear of the subscapularis (LETS), the most important tendinous portion of SSC.

**Methods:**

A total of 233 patients who underwent arthroscopic repair for anterior and superior cuff tears between January 2018 to September 2019 were retrospectively reviewed. The provocative test we have coined as the “scissors sign” and the other related clinical tests (i.e., belly press, belly off, Napoleon, lift off, internal rotation lag, bear hug tests) were performed preoperatively. Whether the patient has a LETS or the complete tear of the SSC (CTS) was confirmed by arthroscopic findings. Sensitivity, specificity, and areas under the receiver operating characteristic curve were calculated for each test.

**Results:**

In patients who had LETS with or without supraspinatus tear, the scissors sign showed the highest sensitivity (91.4%) with a specificity of 81.6%, positive predictive value (PPV) of 80.2%, and negative predictive value (NPV) of 92.1%. In patients with isolated LETS, the scissors sign also showed the highest sensitivity (90.3%) with a specificity of 81.6%, PPV of 57.1%, and NPV of 96.8%. The scissors sign for the complete tear of the subscapularis (CTS) with or without supraspinatus tear and the isolated CTS had a sensitivity of 73.1 and 75%, respectively.

**Conclusions:**

The scissors sign is a novel provocative test that can be helpful in the diagnosis of subscapularis tears, especially LETS, with its high sensitivity and diagnostic accuracy. In combination with other tests, the scissors sign will be a good screening tool.

**Supplementary Information:**

The online version contains supplementary material available at 10.1186/s12891-022-05621-1.

## Background

Several different physical examination tests and signs have been described to aid in the diagnosis of subscapularis (SSC) tear [1]. Some well-known methods include the belly press test [2], belly off sign [3], internal rotational lag sign (IRLS) [4], Napoleon sign (modified belly press) [5], lift off test [6], and bear hug test [7]. Although these can be very helpful especially when used in combination, these tests have limitations and variable sensitivity [3, 4, 7–9], and there has not been any sole reliable test to detect the leading-edge tear of subscapularis (LETS), the most important tendinous portion of SSC, in particular [7, 10–13].

The incidence of SSC tears has been reported to be as high as 50% of arthroscopic repairs [7, 14–16], with the incidence of LETS being the highest among the group [17]. One of the possible symptoms of an SSC tear or pathology includes vague, diffuse anterior shoulder pain [18]. Localization of the LETS to only the upper portion of the SSC makes detection by physical examination or magnetic resonance imaging (MRI) difficult [10, 16, 19, 20]. Smaller partial tears can be missed on MRI scans [16, 21]. Distinguishing the SSC tear from possible coexisting pathologies, such as supraspinatus (SST) tears, can also be difficult. Furthermore, the biceps tendon can be subluxated to disturb the visualization of the localized SSC tear. Also, the pathologies of the biceps tendons may have anterior shoulder pain, which sometimes cannot be distinguished with SSC tear [22]. Therefore, some of these tears are seen incidentally during shoulder arthroscopy procedures initially aimed at addressing other pathologies. Depending on the degree of the tear, patients could benefit from the repair of SSC tears in many cases [17]. Thus, it will be very useful to be able to detect these lesions more reliably before the planned procedures, clinically determining how likely these lesions contribute to the symptoms.

Gerber and Krushell reported on patients who sustained isolated and complete SSC tears following forceful external rotational with the arm adducted at the side [6]. However, the greatest strain exerted by the humeral head on the SSC tendon biomechanically is likely when the maximal external rotational force was applied with the arm abducted at approximately 60° [23–26]. Therefore, a provocative test or sign that can reproduce this exact mechanism may be more reliable in detecting lesions or pathologies localized to the SSC.

The purpose of this study was first, to introduce a new clinical sign coined as the “scissors sign” as elicited by a provocative test which can be useful in detecting SSC tears, and second, to assess the diagnostic accuracy of this sign confirmed by arthroscopic findings in diagnosing any SSC tear and LETS. Thirdly, we aimed to perform comparisons with other known signs and tests, including an MRI scan. Further analysis was performed to see if any confounding factors, such as coexisting SST tears, influence the diagnostic accuracy of the scissors sign. We hypothesized that the sensitivity, specificity, positive predictive values, and negative predictive values for the scissors sign will be competitive in comparison with those of other tests and signs and that it will be especially useful in detecting the LETS.

## Methods

**Fig. 1:**
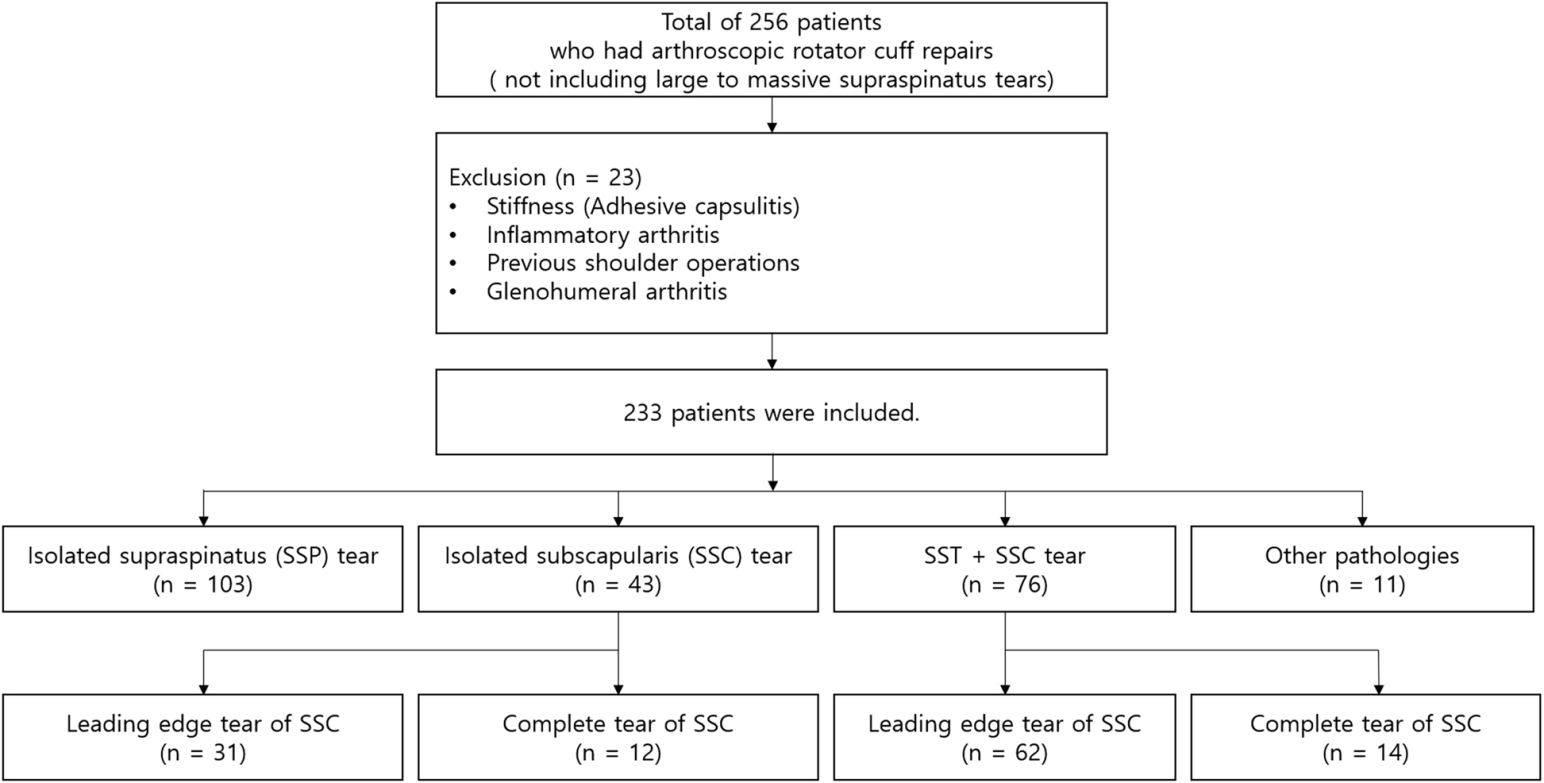
Patient eligibility and allocations according to the arthroscopic findings. LETS included grades I, IIa, IIb and III, while CTS included grades IV and V tears according to the Yoo and Rhee classification.28 SST, supraspinatus; SSC, subscapularis; LETS, leading edge tear of subscapularis; CTS, complete tear of subscapularis

This study was a retrospective cohort study which was approved by the institutional review board. Patients who had arthroscopic rotator cuff repair for superior SST full thickness tears and/or SSC full thickness tears between January 2018 to September 2019 were included. Large or massive tears (> 3 cm) involving the infraspinatus were excluded to limit possible confounding factors and to retain a focus on more anterior pathologies of the rotator cuff [27]. We also excluded those with concomitant shoulder conditions that may significantly affect our results, particularly those with significant stiffness, inflammatory arthritis, previous shoulder operations, and arthritic changes in the glenohumeral joints. After exclusion, 233 patients were included in the study (Fig. 1). Preoperative documentation of physical examinations, including the various tests on specifying SSC tears, were obtained. These were correlated with the documented intraoperative arthroscopic findings. All the physical examination findings were performed or re-checked by the senior author (Y.G.R.), and all operative findings were recorded by the same senior author. The physical examination included the range of motion, scissors sign, belly press test, belly off sign, Napoleon test, lift off test, IRLS, and bear hug test. Preoperative MRI scans and their reports were also reviewed to be included in the comparison. A 3.0-T imaging unit (Achieva; Philips Medical Systems) with a dedicated shoulder coil was utilized. The MRI scans were obtained with the following sequences from the 3.0-T system: axial fat-suppressed protondensity-weighted (PDW) (field of view [FOV], 140 · 140 mm; TR/TE [repetition time/echo time], 4200/30; flip angle, 90; matrix, 320 · 240; section thickness, 2.0 mm; and intersection gap, 0.2 mm), axial turbo spin echo (TSE) T2-weighted (FOV, 140 · 140 mm; TR/TE, 3600 to 4000/80; matrix, 256 · 255; section thickness, 2.0 mm; and intersection gap, 0.2 mm), oblique coronal TSE T1-weighted (FOV, 140 · 140 mm; TR/ TE, 500/10; matrix, 320 · 250; section thickness, 2.0 mm; and intersection gap, 0.5 mm), oblique coronal TSE T2-weighted (FOV, 140 · 140 mm; TR/TE, 3500 to 4000/80; matrix, 350 · 248; section thickness, 2.0 mm; and intersection gap, 0.2 mm), oblique coronal PDW (FOV, 140 · 140 mm; TR/TE, 3500/30; matrix, 320 · 250; section thickness, 2.0 mm; and intersection gap, 0.2 mm), oblique sagittal TSE T2-weighted (FOV, 140 ·140 mm; TR/TE, 5400 to 6000/80; matrix, 328 · 240; section thickness, 2.0 mm; and intersection gap, 0.5 mm). In some cases, axial T1-weighted, coronal fatsuppressed T2-weighted, or sagittal images were also obtained. The MRI scans were interpreted by a radiologist who had more than 10 years of experience on the musculoskeletal disorders.

### Scissors sign

**Fig. 2:**
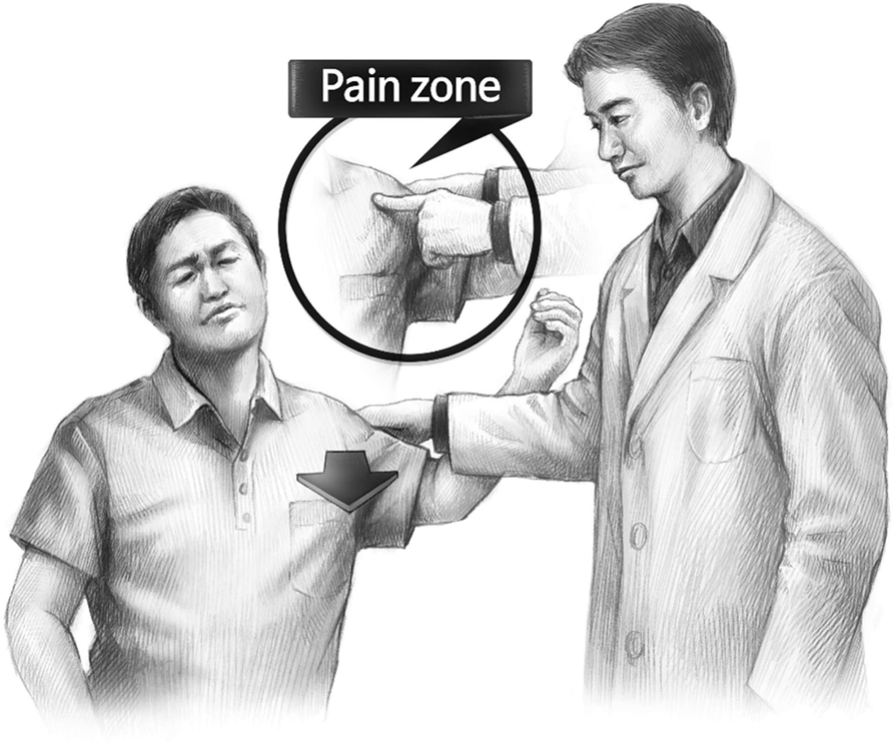
The scissoring effect of the examiner’s arm creates anterior translational force (arrow) onto the humeral head at around 60° of shoulder abduction, which may provoke the injured or pathological part of the subscapularis

The patient was seated comfortably on a chair, and the shoulder was checked for the range of motion first to rule out stiffness. The examiner stood on the patient’s affected side, held the upper limb, and placed the shoulder in abduction by about 60°. At the same time, the examiner’s arm was placed diagonally across over the patient’s arm (as if the limbs are “scissoring” one another), so that the examiner’s palm can cup around and hold the posterior proximal humerus close to the joint, while the patient’s forearm and hand are held back behind the examiner’s arm. In this position, the examiner’s hand and arm can create rotational and anterior translational force by externally rotating the patient’s shoulder, and at the same time, pushing or levering the humeral head forward to perform the provocative test, as depicted in Fig. 2. The patients were told to remain relaxed before the passive stretch test of the shoulder. When the patient reported anterior shoulder pain during the maneuver, the provocative test or scissors sign is considered positive or abnormal, indicating a possible SSC tear or pathology. If pain is not felt at all or felt in the posterior aspect of the shoulder or elsewhere, the test or sign is considered negative.

The sensitivity and specificity of the scissors sign were evaluated in correlation with actual arthroscopic findings and confirmed diagnoses. Diagnostic accuracy and receiver operating characteristic (ROC) curves were calculated. Similarly, these values were also evaluated for other tests (including MRI) and signs of SSC tear, and comparisons with the scissors sign were made. Analysis were also performed for the control group with isolated SST tear, the SSC tears with or without SST tears, and the patients with other concomitant pathologies including biceps lesions, calcific tendinitis, SLAP (superior labrum anterior to posterior) lesions. The video clip demonstrating the examination to elicit the scissors sign in a patient with pathological subscapularis was provided as a supplementary file.

### Arthroscopic inspection

After anesthetic induction, the patient sat up to 60-70° and was appropriately positioned and stabilized in the beach-chair position. After preparation and draping of the shoulder in a sterile manner, a standard posterior portal into the intra-articular space was created using a trocar. Standard arthroscopic observations were performed on structures in the shoulder, including the long head of the biceps anchor, labrum, glenohumeral articular surfaces, and rotator cuff. The SSC and its insertion or footprint could be examined better with the shoulder positioned at 45° abduction and internal rotation. A 70° scope was used to assist with better visualization, especially if the degree of SSC tear was more extensive and required repair. A separate portal could be created anteriorly through the rotator cuff interval, just above the superior edge of the SSC, to introduce probes and graspers to examine the structures in detail, and clearly define the torn edge of the SSC. If an SSC tear was observed, it was then classified according to the description in Yoo et al. [17] The LETS group included Yoo and Rhee classification types I, IIa, IIb, and III, while the complete tear of the SSC (CTS) group included types IV and V. The groups were further divided into those with or without SST tears for further analysis. Any other relevant pathologies, confirmed by arthroscopy, around the superior labrum anterior to posterior (SLAP), the existence of the biceps lesion, signs of subacromial impingement, or calcific tendinitis were noted. The video clip demonstrating the arthroscopic findings during scissors sign in a patient with pathological subscapularis was provided as a supplementary file.

### Statistical analysis

A chi-square test was performed to determine if the differences in the incidence of other accompanying lesions were significant between the groups. Univariate and multivariate logistic regression analysis were performed to determine if there were significant associations between a positive scissors sign and other accompanying shoulder lesions, reported with 95% confidence intervals. The Statistical Package for the Social Sciences (SPSS) software package (version 25.0; SPSS, Chicago, IL, USA) was used for all statistical analysis.

## Results

A total of 256 patients were identified to be eligible for the retrospective review. After exclusion of the patients who did not satisfy the inclusion and exclusion criteria, 233 patients were finally included in the study. The mean age at the time of surgery was 60.1 ± 8.7 years (range: 21-82 years). The study included 120 men and 113 women. Of the 233 arthroscopic findings of patients, the incidence of isolated SSC tears was 18.5% (*n* = 43), SSC tears with SST tears was 32.6% (*n* = 76), SST tears only without any signs of SSC tear or injury was 44.2% (*n* = 103). The rest of the arthroscopies (4.7%) revealed pathologies other than rotator cuff tears (Fig. 1). The concomitant arthroscopic findings were noted in the Table 1.

**Table 1:** Other pathologic finding

Other pathologic findings	Number
Biceps lesion	108 (46.4%)
Biceps tenotomy	78
Biceps tenodesis	30
Calcific tendinitis on subscapularis	9 (0.04%)
Calcific tendinitis on supraspinatus	4 (0.02%)
SLAP lesion	3 (0.01%)

*SLAP* superior labrum anterior to posterior

### Diagnostic values of various tests for an isolated SSC tear

The scissors sign had the highest overall sensitivity at 86.1%, NPV at 93.9%, and diagnostic accuracy at 82.8%. The sensitivity of the scissors sign was also the highest at 90.3% for the LETS subgroup and 75% for the CTS subgroup. The sensitivities of the belly off and bear hug tests were both 75% in the CTS subgroup. The scissors sign had a specificity of 81.6%, PPV of 57.1%, and NPV of 96.8% in the LETS subgroup. The area under the ROC curve was the highest for the scissors sign in the LETS subgroup at 0.860. However, the scissors sign was lower than that of the belly off and bear hug tests in the CTS subgroup. Diagnostic values of each test are shown in Table 2.

**Table 2:** Diagnostic values (95% confidence interval) of various tests and signs for tear of the subscapularis

Tests and Signs	Sensitivity (%)	Specificity (%)	Positive predictive value (%)	Negative predictive value (%)	Diagnostic accuracy (%)	AUC/ROC^**a**^
*LETS*^*b*^ (*N* = 31)						
Scissors	90.3 (79.9-100.0)	81.6 (74.5-88.7)	57.1 (43.3-71.0)	96.8 (93.4-100.0)	83.5	0.860
Belly press	41.9 (24.6-59.3)	89.5 (83.8-95.1)	52.0 (32.4-71.6)	85.0 (78.6-91.4)	79.3	0.657
Belly off	38.7 (21.6-55.9)	92.1 (87.2-97.1)	57.1 (36.0-78.3)	84.7 (78.3-91.0)	80.7	0.654
Napoleon	22.6 (7.9-37.3)	91.2 (86.0-96.4)	41.2 (17.8-64.6)	81.3 (74.5-88.0)	76.6	0.569
Lift off	19.4 (5.4-33.3)	95.6 (91.9-99.4)	54.6 (25.1-84.0)	81.3 (74.7-87.9)	79.3	0.575
IRLS^c^	19.4 (5.4-33.3)	95.6 (91.9-99.4)	54.6 (25.1-84.0)	81.3 (74.7-87.9)	79.3	0.575
Bear hug	45.2 (27.6-62.7)	90.4 (84.9-95.8)	56.0 (36.5-75.5)	85.8 (79.6-92.1)	80.7	0.678
*CTS*^*d*^ (*N* = 12)						
Scissors	75.0 (50.5-99.5)	81.6 (74.5-88.7)	30.0 (13.6-46.4)	96.8 (93.4-100.0)	81.0	0.783
Belly press	41.7 (13.8-69.6)	89.5 (83.8-95.1)	29.4 (7.8-51.1)	93.6 (89.0-98.2)	84.9	0.656
Belly off	75.0 (50.5-99.5)	92.1 (87.2-97.1)	50.0 (26.9-73.1)	97.2 (94.1-100.0)	90.5	0.836
Napoleon	66.7 (40.0-93.3)	91.2 (86.0-96.4)	44.4 (21.5-67.4)	96.3 (92.7-99.9)	88.9	0.789
Lift off	66.7 (40.0-93.3)	95.6 (91.9-99.4)	61.5 (35.1-88.0)	96.5 (93.1-99.9)	92.9	0.770
IRLS^c^	50.0 (21.7-78.3)	95.6 (91.9-99.4)	54.6 (25.1-84.0)	94.8 (90.7-98.8)	91.3	0.728
Bear hug	75.0 (50.5-99.5)	90.4 (84.9-95.8)	45.0 (23.2-66.8)	97.2 (94.0-100.0)	88.9	0.827

^a^*AUC/ROC* area under the curve and receiver operating characteristics

^b^*LETS* leading edge tear of the subscapularis

^c^*IRLS* internal rotational lag sign

^d^*CTS* compete tear of the subscapularis

### Overall diagnostic values of various tests for the SSC tear with or without the SST tear

Overall, regardless of the presence of the SST tear, the scissors sign had the highest sensitivity in diagnosing SSC tears at 87.4%, with the AUC/ROC also being the highest at 0.845 (Table 3). Its sensitivity was 91.4% in the LETS subgroup, with a specificity of 81.6%, PPV of 80.2%, and NPV of 92.1%, with an AUC/ROC of 0.865 (Table 4). All other tests showed sensitivities of less than 50%. For the CTS subgroup, even though its sensitivity was the highest at 73.1%, the AUC/ROC was lower than those of the belly off, Napoleon, and bear hug tests.

**Table 3:** Overall diagnostic values (95% confidence interval) of various tests and signs for subscapularis tear

Tests and Signs	Sensitivity (%)	Specificity (%)	Positive predictive value (%)	Negative predictive value (%)	Diagnostic accuracy (%)	AUC/ROC^**a**^
Scissors	87.4 (81.4-93.4)	81.6 (74.5-88.7)	83.2 (76.7-89.8)	86.1 (79.6-92.6)	84.6	0.845
Belly press	37.0 (28.3-45.7)	89.5 (83.8-95.1)	78.6 (67.8-89.3)	57.6 (50.4-64.9)	62.7	0.632
Belly off	42.0 (33.2-50.9)	92.1 (87.2-97.1)	84.8 (75.6-93.9)	60.3 (53.1-67.6)	66.5	0.671
Napoleon	36.1 (27.5-44.8)	91.2 (86.0-96.4)	81.1 (70.6-91.7)	57.8 (50.6-65.0)	63.1	0.637
Lift off	27.7 (19.7-35.8)	95.6 (91.9-99.4)	86.8 (76.1-97.6)	55.9 (48.9-62.9)	60.9	0.617
IRLS^b^	18.5 (11.5-25.5)	95.6 (91.9-99.4)	81.5 (66.8-96.1)	52.9 (46.1-59.7)	56.2	0.571
Bear hug	45.4 (36.4-54.3)	90.4 (84.9-95.8)	83.1 (74.0-92.2)	61.3 (54.0-68.7)	67.4	0.679

^a^*AUC/ROC* area under the curve and receiver operating characteristics

^b^*IRLS* internal rotational lag sign

**Table 4:** Overall diagnostic values (95% confidence interval) of tests and signs for tear of subscapularis

Tests and Signs	Sensitivity (%)	Specificity (%)	Positive predictive value (%)	Negative predictive value (%)	Diagnostic accuracy (%)	AUC/ROC^**a**^
*LETS*^*b*^ (*N* = 93)						
Scissors	91.4 (85.7-97.1)	81.6 (74.9-88.9)	80.2 (72.6-87.8)	92.1 (87.1-97.4)	86.0	0.865
Belly press	33.3 (23.8-42.9)	89.5 (83.8-95.1)	72.1 (58.7-85.5)	62.2 (54.8-69.6)	64.3	0.614
Belly off	33.3 (23.8-42.9)	92.1(87.2-97.1)	77.5 (64.6-90.4)	62.9 (55.5-70.2)	65.7	0.627
Napoleon	28.0 (18.8-37.1)	91.2 (86.0-96.4)	72.2 (57.6-86.9)	60.8 (53.5-68.1)	62.8	0.596
Lift off	22.6 (14.1-31.1)	95.6 (91.9-99.4)	80.8 (65.6-95.9)	60.2 (53.1-67.4)	62.8	0.591
IRLS^c^	14.0 (6.9-21.0)	95.6 (91.9-93.9)	72.2 (51.5-92.9)	57.7 (50.6-64.7)	58.9	0.548
Bear hug	39.8 (29.8-49.7)	90.4 (84.9-95.8)	77.1 (65.2-89.0)	64.8 (57.4-72.2)	67.6	0.651
*CTS*^*d*^ (*N* = 26)						
Scissors	73.1 (56.0-90.1)	81.6 (74.5-88.7)	47.5 (32.0-63.0)	93.0 (88.0-98.0)	80.0	0.773
Belly press	50.0 (30.8-69.2)	89.5 (83.8-95.1)	52.0 (32.4-71.6)	88.7 (82.9-94.5)	82.1	0.697
Belly off	73.1 (56.0-90.1)	92.1 (87.2-97.1)	67.9 (50.6-85.2)	93.8 (89.3-98.2)	88.6	0.826
Napoleon	65.4 (47.1-83.7)	91.2 (86.0-96.4)	63.0 (44.7-81.2)	82.0 (77.0-87.1)	86.4	0.783
Lift off	46.2 (27.0-65.3)	95.6 (91.9-99.4)	70.6 (48.9-92.2)	88.6 (83.0-94.2)	86.4	0.709
IRLS^c^	34.6 (16.3-52.9)	95.6 (91.9-99.4)	64.3 (39.2-89.4)	86.5 (80.5-92.5)	84.3	0.651
Bear hug	65.4 (47.1-83.7)	90.4(84.9-95.8)	60.7 (42.6-78.8)	92.0 (86.9-97.0)	85.7	0.779

^a^*AUC/ROC* area under the curve and receiver operating characteristics

^b^*LETS* leading edge tear of the subscapularis

^c^*IRLS* internal rotational lag sign

^d^*CTS* complete tear of subscapularis

### Diagnostic values of various tests for the SSC tear in the presence of SST tear

When the subgroup of SSC tear in the presence of SST tear (*n* = 76) was compared with the control group without SSC tear (SST tear only, and other pathologies; *n* = 114), the scissors sign had the highest sensitivity at 88.2%, along with the highest NPV and diagnostic accuracy at 91.2 and 84.2%, respectively, but the specificity of other tests such as the lift off and the IRLS was higher at 95.6% for both. The AUC/ROC was the highest for the scissors sign at 0.849. The scissors sign had the highest sensitivity at 91.9 and 71.4% for the LETS and CTS subgroups, respectively. It also had the highest NPV and diagnostic accuracy for the LETS subgroup at 94.9 and 85.2%, respectively. The AUC/ROC was highest for the scissors sign in the LETS subgroup at 0.868, while it was the highest for the belly off test in the CTS subgroup at 0.818.

### Overall diagnostic values of the MRI scan in diagnosing the SSC tear

As reviewed and reported by radiologists for cases in the presence of SST tears, MRI assessment had a sensitivity of 85.7% for the CTS and 62.5% for the LETS subgroup. When patients with isolated SSC tears were analyzed further, the sensitivity of MRI was 75.0% for the CTS and 66.7% for the LETS subgroups.

### Other pathologies associated with a positive scissors sign

On multivariate logistic regression analysis of our patient cohort, calcific tendinitis in the SSC was significantly associated with the positive scissors sign (*p* <  0.001). SST tears and lesions of the long head of the biceps were statistically irrelevant variables to the scissors sign (Table 5).

**Table 5:** Multivariable logistic regression analysis on other variables for positive scissors sign

	Multivariable analysis	
	OR^a^ (95% CI^b^)	*p* value
Supraspinatus tear	0.98 (0.38-2.55)	0.97
Biceps lesion	1.61 (0.72-3.61)	0.25
Calcific tendinitis in subscapularis	24.84 (22.75-45.07)	< 0.001

^a^*OR* odds ratio

^b^*CI* confidence interval

## Discussion

Our study demonstrated that the scissors sign had a higher sensitivity (91.4% overall, 91.9% in the presence of SST tears, and 90.3% in isolated SSC tears) for the diagnosis of LETS than other physical tests and signs. However, the sensitivity of the scissors sign was similar to that of some other tests (73.1% overall, 71.4% in the presence of SST tears, 75.0% in isolated SSC tears) for the diagnosis of CTS. Therefore, the novel provocative test could be a useful tool to aid in the diagnosis of LETS.

LETS can be difficult to diagnose preoperatively since there have been no reliable diagnostic tests or signs to detect the lesion, and it can sometimes be missed during arthroscopy as well [3, 17, 21, 28, 29]. The MRI scan can assist with the diagnosis but it is also well-known that smaller tears of less than 50% width can be easily missed on MRI due to its low sensitivity of 56% [21]. This was similarly seen in our study, with the MRI sensitivity for LETS being 62.5% in the presence of SST tears, and 66.7% for isolated SSC tears. Notably, the sensitivity of MRI was better for CTS. Studler et al. also demonstrated relatively low sensitivity for MRI when diagnosing SSC tears in association with cortical irregularities or cysts in the lesser tuberosity at 44 and 21%, respectively [30]. However, these findings were not investigated in our study. Overall, the MRI scan, which is often extremely useful in investigating rotator cuff tears among other shoulder pathologies, may not be as reliable in detecting LETS.

LETS has been described as a hidden lesion with its diagnostic difficulty despite being a source of shoulder pain and dysfunction at times [31]. The patients often report non-specific anterior shoulder pain [17, 32]. As most SSC tests aim to detect weakness in internal rotation secondary to the disruption of the tendon, smaller or partial tears such as LETS may not always be found clinically. The inadequacy and variabilities of the diagnostic values for the well-known tests and signs were demonstrated in our study, with most of their sensitivities being under 50%. The Napoleon test’s positive findings are directly and positively correlated with the size or the degree of the tear [11], and the lift-off test does not become positive until at least 75% of the SSC is torn [7]. Unfortunately, due to the persistent symptoms and possible propagation of the tear [17], the missed cases of LETS may lead to delayed surgical treatment [17, 32]. The reported incidence of SSC tears can be as high as 50% [7, 14, 15], and the incidence of LETS is approximately 80% among SSC tears [17]. Therefore, a novel test or sign would be necessary to improve the detection of these relatively common lesions.

Our new provocative test, coined as the “scissors sign”, aims to directly target and stretch the commonly injured upper portion of the SSC attachment where the source of pain is often localized. The humeral head exerts a force directly onto the SSC or its torn area when the shoulder is at the maximum external rotation with an abduction of approximately 60°. This angle, rather than less than 60°or towards 90° or more, is the angle when the SSC may be at its more vulnerable position, resulting in complete or partial tears during strain or traumatic events such as anterior shoulder dislocation [23–26]. Thus, instead of relying on the reduced strength during active contraction due to the disruption of tendon attachment, as seen in many other well-known tests and signs, the scissors sign is elicited by the provocative maneuver, which directly stimulates the pathological area of concern. The stretching of the pathological or torn area is done passively with minimal or no active contraction of the SSC. Thus, even small or partial tears, such as the LETS, may be detected without the remaining intact tendon’s compensation to hide the lesion. Further biomechanical studies, possibly using electromyography, are warranted to confirm this theory.

Because of the provocative maneuver involved in eliciting the scissors sign, it could also let the surgeons know whether the existing tear, especially the LETS, is symptomatic for the patient or not. Although the presumed diagnosis of LETS may be made by other modes of investigation, such as MRI or ultrasonography, the minor degree of the tears that could be asymptomatic may not necessarily require surgical treatment; therefore, it would be useful to know whether or not these smaller tears can be provoked to elicit the pain before commencing the planned arthroscopy.

On the other hand, when the SSC is completely detached from its insertion, as seen in the Yoo and Rhee classifications IV and V [17], there may not be any tension left of the remaining intact structure during the passive stretch maneuver, possibly resulting in less pain. For this reason, these types of tears may have a lower incidence of a positive scissors sign, as reflected in our study, with the sensitivity for the CTS group being lower than that of the LETS group. In these situations, other well-known tests that rely on reduced strengths may be more appropriate in approaching the diagnosis. Thus, even though the scissors sign proved to be a good screening tool on its own, a more accurate diagnosis of LETS is possible if it is used in combination with other clinical tests and signs that also have high specificity.

Apart from relying on the mostly or partly intact SSC tendon, the lack of passive range of motion could limit the scissors sign as well. If the stiffness in the shoulder with or without pain is significant, the patient’s inability to place the hand behind the back may limit tests such as lift-off and IRLS. Similarly, the painful stretch of the anterior capsule created by the external rotational force while trying to elicit the scissors sign can lead to a false positive test. This is why the patients who demonstrated stiffness by having markedly reduced passive motion with forward elevation and external rotation were excluded from this study. These patients usually had diagnoses of arthrosis or shoulder stiffness. Therefore, the scissors sign is not a good tool for detecting SSC tears in the presence of any stiffness in the shoulder joint.

The false positive results of the scissors sign may also arise from pathologies other than the SSC tear, including the anterior SST tear, the long head of the biceps lesion or the SLAP, and calcific tendinitis of the SSC. These pathologies were not excluded from the study, but the multivariable logistic regression test was performed to demonstrate that the SST tear (*p* = 0.97) and biceps lesions (*p* = 0.25) were not significantly associated with the positivity of the scissors sign. Although this meant that the scissors sign was extremely useful even in the presence of SST tears, it also demonstrated that it could be strongly positive in the presence of calcific tendinitis. Therefore, if there is any radiological or clinical suspicion of calcific tendinitis in SSC, the scissors sign could be positive with this pathology instead of any SSC tear. Further studies are warranted to determine the usefulness of the scissors sign in detecting calcific tendinitis in SSC.

### Limitations

The physical examinations were confirmed and recorded by a single senior surgeon, which may show consistency but may also potentially lead to a less objective assessment. Surgeon bias could occur. Thus, further investigation into the inter-observer and intra-observer reliability of the scissors sign would be useful. Another limitation is the relatively small number of patients for some of the subgroups. Although we did the multivariate logistic regression analysis, and found that the calcific tendinitis in the SSC was significantly associated with the positive scissors sign, the number of patients with calcific tendinitis (*n* = 9) was too small to deduce conclusion. Also. the isolated SSC tear subgroups were significantly smaller in comparison to other subgroups. Therefore, a larger study that looks at isolated SSC tears and their subgroups may be warranted. Lastly, this study did not investigate whether the positive scissors sign disappeared after SSC repair. Thus, a follow-up study assessing postoperative patients with the same maneuver would be interesting.

## Conclusions

The scissors sign is a novel provocative test that can be helpful in the diagnosis of SSC tears, especially LETS, with its relatively high sensitivity and diagnostic accuracy in comparison with other well-known tests. In combination with other tests, the scissors sign will be a good screening tool.

## Supplementary Information

**Additional file 1.**

**Additional file 2.**

## Data Availability

The datasets used and/or analysed during the current study are available from the corresponding author on reasonable request.

## Abbreviations

AUC/ROC: Area under the curve and receiver operating characteristics
CTS: Complete tear of the subscapularis
IRLS: Internal rotation lag sign
LETS: Leading edge tear of the subscapularis
MRI: Magnetic resonance imaging
NPV: Negative predictive value
PPV: Positive predictive value
SLAP: Superior labrum anterior to posterior
SSC: Subscapularis
SST: Supraspinatus
